# Global DNA methylation changes spanning puberty are near predicted estrogen-responsive genes and enriched for genes involved in endocrine and immune processes

**DOI:** 10.1186/s13148-018-0491-2

**Published:** 2018-05-09

**Authors:** Emma E. Thompson, Jessie Nicodemus-Johnson, Kyung Won Kim, James E. Gern, Daniel J. Jackson, Robert F. Lemanske, Carole Ober

**Affiliations:** 1 Department of Human Genetics, The University of Chicago, 920 E 58th St, CLSC Room 501, Chicago, IL 60637 USA; 2 School of Medicine and Public Health, University of Wisconsin-Madison, Madison, WI USA; 3 Department of Pediatrics, Section of Allergy, Immunology and Rheumatology, University of Wisconsin School of Medicine and Public Health-Madison, Madison, WI USA; 4 Department of Obstetrics and Gynecology, The University of Chicago, Chicago, IL USA; 5 Present address: Research and Development, USANA Health Sciences Inc, Salt Lake City, Utah USA; 6 Present address: Department of Pediatrics, Yonsei University College of Medicine, Seoul, South Korea

**Keywords:** Epigenetics, Puberty, Differential methylation, Estrogen, Androgen, Immune response

## Abstract

**Background:**

The changes that occur during puberty have been implicated in susceptibility to a wide range of diseases later in life, many of which are characterized by sex-specific differences in prevalence. Both genetic and environmental factors have been associated with the onset or delay of puberty, and recent evidence has suggested a role for epigenetic changes in the initiation of puberty as well.

**Objective:**

To identify global DNA methylation changes that arise across the window of puberty in girls and boys.

**Methods:**

Genome-wide DNA methylation levels were measured using the Infinium 450K array. We focused our studies on peripheral blood mononuclear cells (PBMCs) from 30 girls and 25 boys pre- and post-puberty (8 and 14 years, respectively), in whom puberty status was confirmed by Tanner staging.

**Results:**

Our study revealed 347 differentially methylated probes (DMPs) in females and 50 DMPs in males between the ages of 8 and 14 years (FDR 5%). The female DMPs were in or near 312 unique genes, which were over-represented for having high affinity estrogen response elements (permutation *P* < 2.0 × 10^−6^), suggesting that some of the effects of estrogen signaling in puberty are modified through epigenetic mechanisms. Ingenuity Pathway Analysis (IPA) of the 312 genes near female puberty DMPs revealed significant networks enriched for immune and inflammatory responses as well as reproductive hormone signaling. Finally, analysis of gene expression in the female PBMCs collected at 14 years revealed modules of correlated transcripts that were enriched for immune and reproductive system functions, and include genes that are responsive to estrogen and androgen receptor signaling. The male DMPs were in or near 48 unique genes, which were enriched for adrenaline and noradrenaline biosynthesis (Enrichr *P* = 0.021), with no significant networks identified. Additionally, no modules were identified using post-puberty gene expression levels in males.

**Conclusion:**

Epigenetic changes spanning the window of puberty in females may be responsive to or modify hormonal changes that occur during this time and potentially contribute to sex-specific differences in immune-mediated and endocrine diseases later in life.

**Electronic supplementary material:**

The online version of this article (10.1186/s13148-018-0491-2) contains supplementary material, which is available to authorized users.

## Background

Many anatomical and physiological differences between boys and girls emerge around the time of puberty, a period marked by considerable metabolic and hormonal change as well as dynamic physiologic transitions. This period is characterized by shifts in male and female sex steroid hormone production [1], as well as sex disparities in the onset and remission of asthma [2, 3] and the development of autoimmune diseases and cardiometabolic risk factors, such as lipid profiles [4, 5], blood pressure [6, 7], and insulin resistance [8], among others.

In fact, the hormonal, immune and metabolic changes that occur during and following puberty have been implicated in susceptibility to a wide range of diseases later in life that differ in prevalence, age of onset, and/or severity between men and women [reviewed in ref. [9]]. It is possible, therefore, that puberty-associated hormonal changes result in profound effects on immune processes [3, 10] and could ultimately contribute to lifelong sex-specific risks for immune-mediated, cardiometabolic and endocrine diseases. Although the contribution of genetic factors to the onset of puberty is well established [11–13], only recently have epigenetic processes in the timing and control of puberty been reported. For example, an association was reported between LINE-1 methylation in peripheral blood cells from 9-year-old girls and decreased odds of experiencing menarche by age 12 [14]. Recently, a genome-wide methylation study in peripheral blood cells from 51 children (20 girls and 31 boys) sampled pre- and post-puberty identified 457 CpGs associated with pubertal age in the combined sexes [15]. Ninety-four of these CpGs predicted puberty status among all samples, and another set of 133 CpGs among boys (but not girls) were associated with circulating reproductive hormone levels. However, because boys and girls were analyzed together in this study, little is still known about epigenetic changes that arise during the window of puberty in males and females, which would differ if these changes are linked to the extreme dimorphism that arises during this period.

We undertook this study to identify global changes in an epigenetic mark, DNA methylation, that occur across the window of puberty in males and females separately and then characterize the genes and pathways associated with these epigenetic changes. We present here the results of a study of methylation patterns in DNA from peripheral blood mononuclear cells (PBMCs) collected pre- and post-puberty (8 and 14 years, respectively) from 30 girl and 25 boy participants in the Childhood Origins of ASThma (COAST) birth cohort study [16]. Our results show striking differences between boys and girls and suggest that epigenetic changes occurring between the ages of 8 and 14 in girls may contribute to estrogen, endocrine, and immune signaling pathways, and ultimately play a role in sex-specific differences in susceptibility to immune-mediated and endocrine diseases later in life.

## Methods

### Sample composition

PBMCs were available for 100 children (50 boys, 50 girls) in the COAST study [16] at both ages 8 and 14 years. White blood cell differentials were performed at both ages, as previously described [17]; two individuals with missing differentials at either age were removed. Pubertal status was assessed by Tanner staging [18, 19]. Children who were not pre-puberty at age 8 (one boy, nine girls) or post-puberty at age 14 (18 boys, three girls) were removed, leaving 55 children for analysis of paired samples (25 boys, 30 girls).

The study was approved by the University of Wisconsin Human Subjects Committee and The University of Chicago Institutional Review Board.

### Sample processing and analysis

PBMCs were stored at − 80 °C in cell culture freezing media (ThermoFisher Scientific, Waltham, MA) after collection. DNA for methylation studies was extracted from thawed PBMCs using the Qiagen AllPrep kit (QIAGEN, Valencia, CA). Genome-wide DNA methylation was assessed using the Illumina Infinium Human Methylation 450k BeadChip (Illumina, San Diego, CA) at the University of Chicago Functional Genomics Facility (UC-FGF). Data were processed using Minfi [20]; Infinium type I and type II probe bias were corrected using SWAN [21]. Raw probe values were corrected for color imbalance and background by control normalization. We removed probes that map to the sex chromosomes or to more than one location in a bisulfite-converted genome, had detection *P* values greater than 0.01 in 75% of samples, or overlapped with known single nucleotide polymorphisms (SNPs). Data quality was assessed using principal components analysis (PCA) [22], which identified chip and plate location as potential confounding variables. These effects were removed using ComBat [23]. Methylation levels are reported as β values at each CpG site, which is the fraction of signal obtained from the methylated beads over the sum of methylated and unmethylated bead signals. The Infinium HumanMethylation450 Manifest was used to generate chromosome coordinates based on hg19.

RNA for gene expression studies was isolated from PBMCs collected at the 14-year (post-puberty) time point and assessed using the Illumina Human HT-12 v4 array at the UC-FGF. RNA was not available at the 8-year (pre-puberty) time point. Probe level raw intensity values across arrays were normalized using quantile normalization, and background-corrected normalized expression values were obtained for each probe using the R package lumi [24]. Probes that were indistinguishable from background intensity (*P* < 0.01), contained more than one HapMap SNP, or mapped to multiple locations in the genome [25] were removed; 25,892 of the 47,265 transcripts present on the array remained after this step. The median probe intensity was used to represent the transcriptional abundance of each gene.

Extraction batch, chip, RNA concentration, and RNA quality were identified as potential confounders by PCA analysis of the gene expression data. The effects of batch and chip were removed using ComBat, and the effects of the quantitative variables (RNA concentration and quality) were regressed out using linear regression.

### Network analysis

Weighted Gene Correlation Network Analysis (WGCNA) [26] was used to identify modules of correlated genes among the unique genes that were nearest to puberty-associated differentially methylated CpG sites. For this analysis, an FDR cutoff of 10% was used for differential methylation to increase the number of genes, and therefore power, yielding 893 female-specific DMPs, which mapped to 562 unique genes that were detected as expressed on the array, and 124 male-specific DMPs, which mapped to 81 unique genes that were detected as expressed on the array. The genes associated with each WGCNA module were used as input for gene enrichment analyses and IPA for the female samples, but the 81 genes in the males did not cluster into correlated modules of transcription.

### Ingenuity pathway analysis

Using annotation provided by Illumina, the location of each CpG site was mapped to the closest transcription start site (TSS), according to ENSEMBL. Gene lists were interrogated using QIAGEN’s Ingenuity Pathway Analysis (IPA; QIAGEN Redwood City, https://www.qiagenbioinformatics.com/products/ingenuity-pathway-analysis/), and network associations were constructed using the Ingenuity Knowledge Base. Network interactions were limited to those known to occur in primary cells or tissues; all other settings were left as the defaults. The score of each network is based on the network hypergeometric distribution and is calculated with the right-tailed Fisher’s Exact Test to identify over-representation of the genes near DMPs relative to all genes present on the Illumina HT12 v4 array.

### Statistical analyses

Data were analyzed with R software (version 3.3.0) using a 2 × 2 interaction test in limma [27]. A random effects model with individual ID coded as a random effect to account for the paired sample design was used to identify differentially methylated CpG sites in males and females pre- and post-puberty. Race/ethnicity was not a significant covariate and was not included in the model; however, to exclude the possibility of confounding from these subjects, differential methylation analysis was repeated after excluding the five children of non-European ancestry (Table 1 and Additional file 1). Age-specific cell composition (% lymphocytes, % monocytes, and % eosinophils) was not included as a covariate; instead, we looked for effects of cell composition in downstream analyses. All enrichment analyses were conducted using Enrichr (http://amp.pharm.mssm.edu/Enrichr/) [28, 29]. Gene lists (genes nearest DMPs and genes comprising networks identified through WGCNA) were used as input and default settings were used for analysis. Permutations were performed by randomly selecting 312, 198, or 86 genes (for the data presented in Table 2 and Additional file 2, respectively) from the list of 3497 genes with high affinity estrogen receptor binding sites, then comparing the random sample with the observed gene list and recoding how many times (out of 500,000) the number of genes was equal to (or greater than) the observed value (63, 53, or 20, respectively). Correlations between differentially methylated CpGs and expression level of the nearest gene at age 14 were tested using Pearson coefficients as implemented in R.

**Table 1:** Size and ethnic composition of sample

	Males *N* (%)	Females *N* (%)
Sample size	25	30
Race/ethnicity		
European American	23 (92)	27 (90)
African American	2 (8)	1 (3)
Hispanic	0	2 (6)

**Table 2:** Predicted estrogen-responsive genes are over-represented near female puberty-associated DMPs. *P* < 2.0 × 10^−6^ by permutation testing

	No. of genes with high affinity estrogen-responsive elements (%)	No. of all other genes (%)	Total
Near a puberty DMP	63 (20.2)	249 (79.8)	312 (100)
Near a Non-DMP	3434 (14.7)	19,883 (85.3)	23,317 (100)
Total	3497 (14.5)	20,132 (85.5)	23,629 (100)

*DMP*, differentially methylated probe

### Availability of data and materials

The datasets supporting the conclusions of this article will be made available at the time of publication.

## Results

### Identifying differentially methylated CpGs at 8 and 14 years of age

To assess global DNA methylation changes that occur between the ages of 8 and 14, we first identified differentially methylated probes (DMPs) (5% FDR) in the combined sample (*n* = 55 pairs), girls only (*n* = 30 pairs), and boys only (*n* = 25 pairs).

**Fig. 1:**
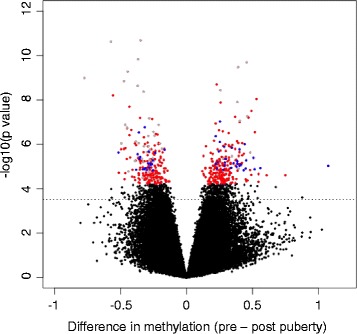
Volcano plot showing differences in methylation between 8 and 14 years of age in males and females combined (DMPs in 55 paired samples). Significant (FDR = 5%) DMPs are shown as non-black circles. Female puberty-associated DMPs (347 in 30 paired female samples) are shown in red, male puberty-associated DMPs (50 DMPs in 25 paired samples) are shown in blue, and CpGs identified as being differentially methylated in the combined sample (48 in 55 paired samples) are shown in gray. The *x* axis shows –log10 *P* values and the *y* axis plots the mean difference in methylation *β* values. The horizontal line denotes significant CpGs at an FDR of 10%

Overall, we detected a total of 445 DMPs: 48 in the combined sample (gray in Fig. 1), 347 unique to girls (shown in red in Fig. 1), and 50 unique to boys (shown in blue in Fig. 1; DMP lists are available in Additional files 3–5). Among the 347 female-specific DMPs, 155 (44.7%) became more methylated and 192 (55.3%) became less methylated between 8 and 14 years of age. Most of these sites (263 of 347) are either in the body of a gene or within 1500 base pairs of a transcription start site (42.5 and 25.3%, respectively), slightly higher than the overall distribution of CpGs on the array (31 and 17%, respectively). In females, the median absolute change in methylation among DMPs was 2.6% (ranging from 1.6 to 10.5%); 9 CpGs (2.5%) had a change greater than 5%. The 347 female-specific DMPs are located in or near 312 unique genes.

Among the 50 male-specific DMPs, 29 (58%) became more methylated and 21 (42%) became less methylated between 8 and 14 years of age. As in the females, most of the sites (37 out of 50) are in the body of a gene or within 1500 base pairs of a transcription start site (40.0 and 34.0%, respectively). In males, the median absolute change in methylation was 3.2% (ranging from 2.6 to 6.9%); 5 CpGs (10%) had a change greater than 5%. The 50 male-specific DMPs are in or near 48 unique genes.

These results were not influenced by the inclusion of five children of non-European ancestry (Table 1). The beta values of the 347 DMPs (*N* = 55 vs 50 pairs) were significantly correlated between analyses with and without the five non-European individuals (Pearson correlation *r* = 0.82, Additional file 1). Therefore, all subsequent analyses include participants of all ancestries to maximize sample sizes.

### Genes with high affinity estrogen response elements are over-represented among genes near female DMPs

Given the central role of estrogen in the developmental and reproductive changes that accompany puberty, we first asked whether genes near female puberty-associated DMPs were over-represented among potential estrogen-responsive genes. For this analysis, we used a list of genes from published studies revealing high-affinity genome-wide estrogen response elements [30] to represent predicted estrogen response genes (Additional file 6). A comparison of those genes to the genes nearest the 312 female puberty-associated DMPs revealed a significant excess of genes near female puberty-associated DMPs among predicted estrogen response genes (*n* = 62) compared to genes nearest to all CpGs on the array (Permutation *P* < 2.0 × 10^−6^; Table 2). These results suggest that at least some of the effects of estrogen signaling in puberty are modified through epigenetic mechanisms. As expected, there was no such pattern in the male-specific DMPs (*P* = 0.57; data not shown).

We next evaluated whether genes near female puberty-associated DMPs were functionally related to one another. To identify candidate pathways, we used Ingenuity Pathway Analysis (IPA) to construct protein-protein interaction networks using the list of 312 genes as input (Additional file 7). Remarkably, the genes nearest to each of the 347 female puberty-associated DMPs formed four significant networks that were enriched for genes implicated in pubertal timing and initiation, and endocrine system development using Enrichr (Table 3). For example, network 1 (score = 47) included 26 genes enriched for phosphatidylinositol signaling (*P* = 0.0064), which plays a key role in the integration of metabolic and neural signals regulating gonadotropin releasing hormone/luteinizing hormone release. Network 2 (score = 41) included 24 genes enriched for fibroblast growth factor (FGF) signaling (*P* = 0.0073). Network 3 (score = 33) included 21 genes enriched for insulin-like growth factor-1 (IGF-1) signaling (*P* = 0.0098), which has been implicated in growth and metabolism during female puberty (and IGF-1 levels are elevated among girls with precocious puberty [31]). Finally, network 4 (score = 32) included 20 genes enriched for estrogen receptor signaling (*P* = 0.001845). The 48 unique genes nearest to the 50 male puberty-associated DMPs were enriched for adrenaline and noradrenaline biosynthesis (Enrichr Adj *P* = 0.021; Panther 2016). Neither the genes nearest to 50 male puberty-associated DMP nor the genes nearest the 48 DMPs shared between males and females formed any significant networks using IPA, possibly due to the small number of genes use as input.

**Table 3:** Enrichment categories of four significant protein-protein interaction networks in females constructed using Ingenuity Pathway Analysis (networks shown in Additional file 7)

Network (IPA Score)	Enrichr results		
	Enrichment category	Database	Adj. *P* value
1 (47)	Phosphatidylinositol signaling TGF-β receptor signaling	KEGG 2016 Panther 2016	0.0064 0.043
2 (41)	FGF signaling pathway	Panther 2016	0.0073
3 (33)	Insulin-like growth factor-1 (IGF-1) signaling	NCI-Nature 2016	0.0098
4 (32)	Validated estrogen receptor alpha network	NCI-Nature 2016	0.0018

Adjusted *P* values were determined by performing the Fisher Exact Test for many random gene sets in order to compute a mean rank and SD from the expected rank for each term in the gene-set library

### Genes near female puberty-associated DMPs are enriched for immune functions and sex hormone receptor signaling

To gain further insight into the processes and networks that are influenced by epigenetic changes during puberty, we measured transcript levels in post-puberty PMBCs collected at the same age 14 visit as those used for the methylation studies. We detected 18,756 transcripts as expressed in these samples, but for this analysis, we focused on the 562 genes near female puberty-associated DMPs and used a systems biology approach as implemented in Weighted Gene Correlation Network Analysis (WGCNA) to identify modules of correlated transcripts in the females. The motivation for this analysis is to identify groups of functional molecules (based on transcript levels) near DMPs that are correlated with one another. WGCNA assigned 284 (50.5%) of these genes into two co-expression modules; the remaining 278 genes showed no correlation structure in the post-puberty samples. The 198 genes in the first module were significantly enriched for T cell receptor signaling (*P* = 0.0038) and activation (*P* = 0.0021) (Table 4) and were associated as a whole with inflammatory and respiratory diseases (IPA *P* = 3.77 × 10^−4^ for both). The second gene expression module consisted of 86 genes enriched for estrogen receptor beta signaling (*P* = 0.0019) and androgen receptor signaling (*P* = 0.0067). There was an over-representation of genes with high affinity estrogen response elements among genes in both modules compared to all other gene transcripts measured (Permutation *P* < 2.0 × 10^−6^ (module 1 and module 2); Additional file 2).

**Table 4:** Modules of correlated transcripts at genes near female puberty-associated DMPs are enriched for immune functions and hormone signaling

WGCNA Module (# of genes)	Pathway enrichment category (Adjusted *P* value)	Database
1 (198)	T cell receptor signaling (*P* = 0.0038) and activation (*P* = 0.0021)	KEGG 2016, BioCarta 2016
	Epidermal growth factor receptor (EGFR) signaling (*P* = 0.029)	Panther 2016
2 (86)	Estrogen receptor beta signaling (*P* = 0.0019)	KEGG 2016
	Androgen receptor signaling (*P* = 0.0067)	

To investigate possible correlations between CpG methylation and gene expression, we further examined the 259 genes detected as expressed on the array (out of the 312 nearest genes). We observed correlations (*P* < 0.05) for 12/259 comparisons (5%), similar to rates reported in other studies [32, 33].

There were no modules of correlated gene expression using the genes nearest male puberty-associated DMPs, again potentially due to the small number of DMPs in the males (*N* = 124 DMPs and 81 unique genes at an FDR of 10%).

### A subset of CpGs predicts pubertal status in COAST children

Almstrup et al. [15] identified a subset of 94 CpGs in peripheral blood cells that predicted pubertal status and 133 CpGs that predicted circulating hormone levels in males in their study of 51 children (31 boys, 20 girls). Using 75 of the 94 puberty-predicting CpGs that were present in our dataset, we classified the COAST samples as pre- or post-puberty using unsupervised hierarchical clustering. Indeed, methylation changes at these 75 CpGs (Additional file 8) predicted pubertal status among the 55 COAST children, with a specificity of 92.7% and a sensitivity of 87.3% (Table 5). A subset of 104 of the 133 CpGs (Additional file 9) that predicted levels of six circulating sex hormones in males in the Almstrup study were present in our data set. Although hormone levels were not measured in the COAST children, the 104 CpGs separated the males on the basis of pubertal status with a specificity of 72.0% and a sensitivity of 96.0%. Curiously, in our study, we see the greatest overlap between the predictive CpGs reported by Almstrup et al. and the female-specific DMPs: 29% (22/75) of the CpGs used to predict puberty status and 27% (28/104) of the CpGs used to predict hormone levels in boys pre- and post-puberty are present among the 347 DMPs specific to females in our study (indicated in Additional files 8 and 9). In contrast, only 4% (3/75) and 2% (2/104) of the predictive CpGs were present among the male-specific DMPs reported here, and 24% (18/75) and 19% (20/104) were among the DMPs shared by girls and boys in this study.

**Table 5:** Performance of predictive CpG sets reported by Almstrup et al. in this study

	Among COAST females (*N* = 30)		Among COAST males (*N* = 25)		Among COAST males and females combined (*N* = 55)	
	Specificity	Sensitivity	Specificity	Sensitivity	Specificity	Sensitivity
94 puberty classifiers (*N* = 75)	83.3%	96.7%	92.0%	92.0%	92.7%	87.3%
133 reproductive hormone^a^ classifiers (*N* = 104)	86.7%	86.2%	72.0%	96.0%	90.9%	90.9%

^a^Follicle stimulating hormone (FSH), luteinizing hormone (LH), anti-Mullerian hormone (AMH), testosterone (T), estradiol (E2), inhibin B

Numbers in parentheses in first column refer to the number of CpGs in each subset reported by Almstrup that are present in this study

### Changes in cell proportions between 8 and 14 years of age are not associated with DMPs

Finally, we assessed whether changes in cell type proportions contributed to the observed puberty-associated DMPs. In fact, in both boys and girls, there were significant differences in lymphocyte (Wilcoxon Rank Test *P* = 0.0036 and *P* = 0.00029, respectively) and monocyte (*P* = 3.05 × 10^−8^ and *P* = 2.67 × 10^−8^, respectively), but not eosinophil (*P* = 0.59 and *P* = 0.49, respectively) proportions between pre- and post-puberty PBMCs. We next examined whether changes in cell proportions were correlated with changes in methylation levels between ages 8 and 14 years. Among females, changes in methylation levels were not correlated with changes in lymphocyte or eosinophil proportions (*P* > 0.14; Spearman correlation test). Among individual CpG sites, methylation level changes at three were correlated with changes in monocyte proportions at an FDR of 5%, but none of the three CpG sites were puberty-associated DMPs in females. Among males, no significant correlations were observed between methylation changes and cell proportion changes pre- and post-puberty. Moreover, cell proportions were not associated with post-puberty transcript levels among the genes assigned to the two WGCNA modules, indicating that the correlations in gene expression post-puberty were not due to differences in cell proportions among subjects.

## Discussion

Changes in DNA methylation can impact transcription of nearby genes and thereby modulate the effects of hormonal fluctuations in cells and tissues on gene expression. To our knowledge, this is the first report of sex-specific changes in methylation patterns across the window of puberty in humans, a period of dynamic change that can carry long-term implications for health and disease. The results of our unbiased, genome-wide study suggest that epigenetic modifications arise during early adolescence, particularly among females, and that many of these changes occur near genes implicated in traits that differ between males and females during or after sexual maturation. These findings may shed light on endocrine, metabolic, and immune disease susceptibility, among others, through either the identification of novel target genes near DMPs or the recognition of epigenetic mechanisms affecting these phenotypes.

**Fig. 2:**
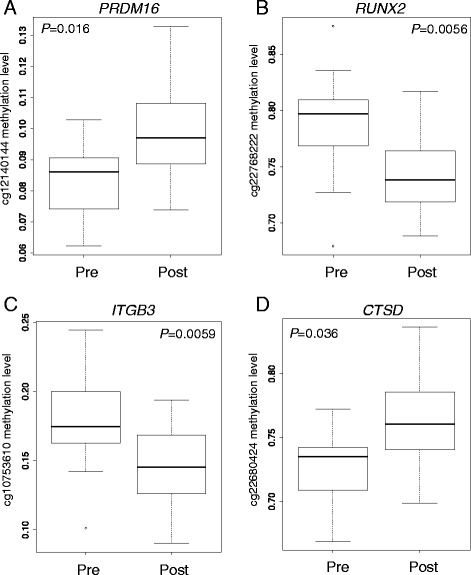
Examples of changes in methylation levels (β values) in four estrogen-responsive genes present in the networks shown in Additional file 7. **a** Methylation levels of cg12140144 (*PRDM16*; network 1), **b** Methylation levels of cg22768222 (*RUNX2*; network 2), **c** Methylation levels of cg10753610 (*ITGB3*; network 3), and **d** Methylation levels of cg22680424 (*CTSD*; network 4)

Our study revealed an over-representation of genes near female DMPs, as well as among correlated modules of gene transcript levels measured post-puberty, that harbor high affinity estrogen response elements. These findings suggest the epigenetic changes that occur over the window of puberty are coordinated with estrogen signaling in females, potentially contributing to long-term health effects. Beyond the critical role estrogen is known to play in female puberty, it also modulates inflammation and immune responses [3, 34–36], influences the severity of a number of autoimmune diseases [37, 38], and is thought to play a role in protection against cardiovascular disease in women [39]. Our findings suggest that DMPs involved in estrogen signaling arise during puberty itself. As such, puberty may represent a unique window with regard to the influx of circulating hormones, and be a time during which girls are particularly sensitive to the effects of DNA methylation changes. These changes and responses to hormones may ultimately influence a wide range of sex-specific traits. For example, *PRDM16*, identified as an estrogen-responsive gene in Network 1 (Fig. 2 and Additional files 6 and 7), controls brown adipose tissue (BAT) differentiation. BAT activity and volume increase during puberty [40], ultimately leading to gains in skeletal musculature consistent with pubertal development; significantly greater changes in BAT volume have been reported in males compared to females [41]. Metabolic and hormonal factors have been proposed to be responsible for this increase, although specific mechanisms have not been elucidated.

Moreover, the predicted estrogen-responsive genes were present as well-connected hubs in four networks. For example, PR/SET Domain 16 (*PRDM16*; network 1), discussed above, is also a regulator of TGF-β signaling; Runt-related transcription factor 2 (*RUNX2*; network 2) is a transcription factor involved in skeletal/bone development; FGF signaling, integrin subunit beta 3 (*ITGB3*; network 3) encodes a cell surface protein with a role in cell migration, adhesion and signaling; and cathepsin D (*CTSD*, network 4) is an A1 peptidase that plays a role in proteolytic activation of hormones and growth factors.

The identification of correlated modules of transcripts for genes involved in immune signaling and sex hormone receptor signaling is intriguing and points to the diverse array of puberty-associated developmental traits in which epigenetics likely plays a role. The enrichment of genes near female puberty-associated DMPs with correlated patterns of expression in protein networks that are centered on these phenotypes is indicative of both the sex-specific nature of many traits (particularly those that are endocrine or immune-related), and the expansive role of epigenetic modifications.

Further evidence for a critical role of DNA methylation during puberty comes from our demonstration that a subset of puberty-specific methylation changes in a combined sample of males and females in the Almstrup study [15] predicted puberty status in COAST children with high sensitivity and specificity. Intriguingly, the greatest proportions of the puberty-predictive CpGs are within the sets of female-specific and shared DMPs in our study. This observation cannot be due simply to the smaller number of DMPs among males than females because the number of DMPs shared with those in the Almstrup study is similar to males and females (48 vs 50, respectively). Despite many parallels between our study and that of Almstrup et al., there are a number of important differences. Both studies evaluated genome-wide methylation patterns via the Illumina 450K array in blood cells collected pre-puberty (~ 8–9 year old) and post-puberty (~ 14–15 year old), and the combined sample sizes were similar (30 girls and 25 boys in our study compared to 20 girls and 31 boys in the Almstrup study). However, Almstrup et al. used peripheral blood leukocytes (PBLs) while we used peripheral blood mononuclear cells (PBMCs), and Almstrup et al. combined both sexes for analysis and used sex as a covariate to identify 457 DMPs in the combined sample, whereas we focused our studies on methylation changes that were unique to boys or to girls and identified sex-specific puberty DMPs (Fig. 1). Although Almstrup et al. did not specifically report sex-specific methylation changes, they state that only data from the boys resulted in significant CpGs when the two groups were analyzed separately, perhaps due to the relatively smaller number of girls in their study. Ultimately, the fact that two predictor sets of CpGs reported in the Almstrup study predict pubertal status in our study likely reflects common pathways involved in pubertal development in both sexes and the robustness and stability of the DNA methylation changes associated with puberty.

The small proportion of correlated DMP-transcript pairs in the post-puberty samples is not entirely unexpected. In fact, previous genome-wide studies have shown that overall few transcripts are correlated with nearby CpG methylation levels [42–44]. Our study was further limited because we did not have RNA for the pre-puberty time point. As a result, we could not test for correlations between changes in DNA methylation and changes in gene expression, a potentially more relevant comparison. In addition, we do not know the time or age at which puberty-associated methylation changes exert their effects on gene expression. It is possible, for example, that relevant changes in transcript abundance due to changes in methylation occur before 14 years of age. Longitudinal sampling over the window of puberty would be required to address these questions.

Our study has other limitations. First, despite discovering many significant DMPs, the size of our sample is relatively small and the observed effect sizes (absolute changes in methylation) were modest. Second, we cannot exclude the possibility that some of the changes in methylation we observe are simply due to age. For example, it is possible that the subset of DMPs that are common to both males and females represent age-specific methylation changes. Although only 1% of the puberty-associated CpG sites have been reported to undergo age-related methylation changes [45, 46] and methylation levels at the CpGs that are known to be associated with aging did not differ between males and females in our study (*P* > 0.05) [45, 46], it remains possible that some of the shared puberty DMPs are due to age-related changes that are unrelated to puberty itself. Third, our study is limited to data from PBMCs. It is possible, and even likely, that different patterns of epigenetic modifications would be present in other tissues.

In conclusion, our results provide evidence for significant female puberty effects on global DNA methylation patterns at CpGs whose nearby genes are enriched for estrogen responsiveness and form networks centered on immune processes and sex hormone signaling, findings that were validated in gene expression studies in the post-puberty period. In addition, two out of four significant protein interaction networks based on genes nearest the puberty DMPs include genes involved in puberty regulation and timing, supporting an important role for epigenetics in this process.

## Conclusions

Genes near differentially methylated CpGs that arise during female puberty are over-represented for estrogen responsiveness and networks focused on endocrine system development as well as immune response. These results suggest that epigenetic changes across the window of puberty are, in part, responsive to the hormonal changes that occur during this time. Ultimately, this research may be useful in identifying genes that potentially contribute to sex-specific diseases later in life.

## Additional files

Additional file 1: Scatterplot illustrating correlation between methylation beta values in all samples (*N* = 55 pairs) and beta values following analysis in subjects of European ancestry only. Beta values of 347 DMPs in females from the full sample are plotted on the *x* axis and beta values from the same DMPs in an analysis using only samples of European ancestry are plotted on the *y* axis. (PDF 41 kb)

Additional file 2: Genes in modules 1 and 2 (as identified by WGCNA) are enriched for predicted estrogen-responsive genes. WGCNA = Weighted gene correlation network analysis. *P* values reflect results of permutation testing as described in Methods. (DOCX 67 kb)

Additional file 3: List of 48 differentially methylated probes in boys and girls, pre- and post-puberty (FDR = 5%). (XLSX 47 kb)

Additional file 4: List of 347 differentially methylated probes unique to girls, pre- and post-puberty (FDR = 5%). (XLSX 82 kb)

Additional file 5: List of 50 differentially methylated probes unique to boys, pre- and post-puberty (FDR = 5%). (XLSX 48 kb)

Additional file 6: List of genes near high affinity estrogen response elements near a female puberty-associated DMP generated from reference [31] and references therein. Genes near high affinity estrogen response elements that are also near female puberty-associated DMPs are listed. (XLSX 30 kb)

Additional file 7: Significant networks of genes associated with female puberty-associated DMCs (from Ingenuity Pathway Analysis). Network 1 score = 47; network 2 score = 41; network 3 score = 33; network 4 score = 32. Genes in blue are shown in more detail in Fig. 2. Shapes shaded in yellow represent genes present in the input list. Solid lines indicate direct interactions between molecules and dotted lines indicate indirect. Arrows indicate the activation of one molecule by another. Legend from https://www.qiagenbioinformatics.com/products/ingenuity-pathway-analysis/. (PDF 3162 kb)

Additional file 8: Set of 75 CpGs present in this study that are a subset of the 99 CpGs predictive of puberty status reported by Almstrup et al. (XLSX 48 kb)

Additional file 9: Set of 104 CpGs present in this study that are a subset of the 133 CpGs predictive of reproductive hormone levels pre- and post-puberty reported by Almstrup et al. (XLSX 49 kb)

## Abbreviations

DMP: Differentially methylated probe
IPA: Ingenuity Pathway Analysis
PBMC: Peripheral blood mononuclear cell
WGCNA: Weighted Gene Correlation Network Analysis

## References

[1] Alonso LC, Rosenfield RL (2002). Oestrogens and puberty. Best Pract Res Clin Endocrinol Metab.

[2] Postma DS (2007). Gender differences in asthma development and progression. Gend Med.

[3] Keselman A, Heller N (2015). Estrogen signaling modulates allergic inflammation and contributes to sex differences in asthma. Front Immunol.

[4] Altwaijri YA, Day RS, Harrist RB, Dwyer JT, Ausman LM, Labarthe DR (2009). Sexual maturation affects diet-blood total cholesterol association in children: Project HeartBeat!. Am J Prev Med.

[5] Morrison JA, Laskarzewski PM, Rauh JL, Brookman R, Mellies M, Frazer M, Khoury P, deGroot I, Kelly K, Glueck CJ (1979). Lipids, lipoproteins, and sexual maturation during adolescence: the Princeton maturation study. Metabolism.

[6] Shankar RR, Eckert GJ, Saha C, Tu W, Pratt JH (2005). The change in blood pressure during pubertal growth. J Clin Endocrinol Metab.

[7] Taittonen L, Uhari M, Turtinen J, Nuutinen M (1997). Change in blood pressure during pubertal insulin resistance. Pediatr Res.

[8] Kelsey MM, Zeitler PS (2016). Insulin resistance of puberty. Curr Diab Rep.

[9] Ober C, Loisel DA, Gilad Y (2008). Sex-specific genetic architecture of human disease. Nat Rev Genet..

[10] Straub RH (2007). The complex role of estrogens in inflammation. Endocr Rev.

[11] Elks CE, Perry JR, Sulem P, Chasman DI, Franceschini N, He C, Lunetta KL, Visser JA, Byrne EM, Cousminer DL (2010). Thirty new loci for age at menarche identified by a meta-analysis of genome-wide association studies. Nat Genet.

[12] Abreu AP, Kaiser UB (2016). Pubertal development and regulation. Lancet Diabetes Endocrinol.

[13] Perry JR, Day F, Elks CE, Sulem P, Thompson DJ, Ferreira T, He C, Chasman DI, Esko T, Thorleifsson G (2014). Parent-of-origin-specific allelic associations among 106 genomic loci for age at menarche. Nature.

[14] Huen K, Harley K, Kogut K, Rauch S, Eskenazi B, Holland N (2016). DNA methylation of LINE-1 and Alu repetitive elements in relation to sex hormones and pubertal timing in Mexican-American children. Pediatr Res.

[15] Almstrup K, Lindhardt Johansen M, Busch AS, Hagen CP, Nielsen JE, Petersen JH, Juul A (2016). Pubertal development in healthy children is mirrored by DNA methylation patterns in peripheral blood. Sci Rep.

[16] Lemanske RF (2002). The childhood origins of asthma (COAST) study. Pediatr Allergy Immunol.

[17] Neaville WA, Tisler C, Bhattacharya A, Anklam K, Gilbertson-White S, Hamilton R, Adler K, Dasilva DF, Roberg KA, Carlson-Dakes KT (2003). Developmental cytokine response profiles and the clinical and immunologic expression of atopy during the first year of life. J Allergy Clin Immunol.

[18] Marshall WA, Tanner JM (1969). Variations in pattern of pubertal changes in girls. Arch Dis Child.

[19] Marshall WA, Tanner JM (1970). Variations in the pattern of pubertal changes in boys. Arch Dis Child.

[20] Aryee MJ, Jaffe AE, Corrada-Bravo H, Ladd-Acosta C, Feinberg AP, Hansen KD, Irizarry RA (2014). Minfi: a flexible and comprehensive Bioconductor package for the analysis of Infinium DNA methylation microarrays. Bioinformatics.

[21] Maksimovic J, Gordon L, Oshlack A (2012). SWAN: subset-quantile within array normalization for illumina infinium HumanMethylation450 BeadChips. Genome Biol.

[22] Leek JT, Scharpf RB, Bravo HC, Simcha D, Langmead B, Johnson WE, Geman D, Baggerly K, Irizarry RA (2010). Tackling the widespread and critical impact of batch effects in high-throughput data. Nat Rev Genet.

[23] Johnson WE, Li C, Rabinovic A (2007). Adjusting batch effects in microarray expression data using empirical Bayes methods. Biostatistics.

[24] Du P, Kibbe WA, Lin SM (2008). lumi: a pipeline for processing Illumina microarray. Bioinformatics.

[25] Nicodemus-Johnson J, Naughton KA, Sudi J, Hogarth K, Naurekas ET, Nicolae DL, Sperling AI, Solway J, White SR, Ober C (2016). Genome-wide methylation study identifies an IL-13-induced epigenetic signature in asthmatic airways. Am J Respir Crit Care Med.

[26] Langfelder P, Horvath S (2008). WGCNA: an R package for weighted correlation network analysis. BMC Bioinformatics.

[27] Ritchie ME, Phipson B, Wu D, Hu Y, Law CW, Shi W, Smyth GK (2015). limma powers differential expression analyses for RNA-sequencing and microarray studies. Nucleic Acids Res.

[28] Chen EY, Tan CM, Kou Y, Duan Q, Wang Z, Meirelles GV, Clark NR, Ma'ayan A (2013). Enrichr: interactive and collaborative HTML5 gene list enrichment analysis tool. BMC Bioinformatics.

[29] Kuleshov MV, Jones MR, Rouillard AD, Fernandez NF, Duan Q, Wang Z, Koplev S, Jenkins SL, Jagodnik KM, Lachmann A (2016). Enrichr: a comprehensive gene set enrichment analysis web server 2016 update. Nucleic Acids Res.

[30] Bourdeau V, Deschenes J, Metivier R, Nagai Y, Nguyen D, Bretschneider N, Gannon F, White JH, Mader S (2004). Genome-wide identification of high-affinity estrogen response elements in human and mouse. Mol Endocrinol.

[31] Sorensen K, Aksglaede L, Petersen JH, Andersson AM, Juul A (2012). Serum IGF1 and insulin levels in girls with normal and precocious puberty. Eur J Endocrinol.

[32] Olsson AH, Volkov P, Bacos K, Dayeh T, Hall E, Nilsson EA, Ladenvall C, Ronn T, Ling C (2014). Genome-wide associations between genetic and epigenetic variation influence mRNA expression and insulin secretion in human pancreatic islets. PLoS Genet.

[33] Medvedeva YA, Khamis AM, Kulakovskiy IV, Ba-Alawi W, Bhuyan MS, Kawaji H, Lassmann T, Harbers M, Forrest AR, Bajic VB, Consortium F (2014). Effects of cytosine methylation on transcription factor binding sites. BMC Genomics.

[34] Draijer C, Hylkema MN, Boorsma CE, Klok PA, Robbe P, Timens W, Postma DS, Greene CM, Melgert BN (2016). Sexual maturation protects against development of lung inflammation through estrogen. Am J Physiol Lung Cell Mol Physiol.

[35] Klein SL, Flanagan KL (2016). Sex differences in immune responses. Nat Rev Immunol.

[36] Lamason R, Zhao P, Rawat R, Davis A, Hall JC, Chae JJ, Agarwal R, Cohen P, Rosen A, Hoffman EP, Nagaraju K (2006). Sexual dimorphism in immune response genes as a function of puberty. BMC Immunol.

[37] Liu HB, Loo KK, Palaszynski K, Ashouri J, Lubahn DB, Voskuhl RR (2003). Estrogen receptor alpha mediates estrogen's immune protection in autoimmune disease. J Immunol.

[38] Khan D, Ansar AS (2015). The immune system is a natural target for estrogen action: opposing effects of estrogen in two prototypical autoimmune diseases. Front Immunol.

[39] Arnal JF, Fontaine C, Billon-Gales A, Favre J, Laurell H, Lenfant F, Gourdy P (2010). Estrogen receptors and endothelium. Arterioscler Thromb Vasc Biol.

[40] Gilsanz V, Hu HH, Kajimura S (2013). Relevance of brown adipose tissue in infancy and adolescence. Pediatr Res.

[41] Gilsanz V, Smith ML, Goodarzian F, Kim M, Wren TA, Hu HH (2012). Changes in brown adipose tissue in boys and girls during childhood and puberty. J Pediatr.

[42] Long MD, Smiraglia DJ, Campbell MJ (2017). The genomic impact of DNA CpG methylation on gene expression; relationships in prostate cancer. Biomol Ther.

[43] Schulz H, Ruppert AK, Herms S, Wolf C, Mirza-Schreiber N, Stegle O, Czamara D, Forstner AJ, Sivalingam S, Schoch S (2017). Genome-wide mapping of genetic determinants influencing DNA methylation and gene expression in human hippocampus. Nat Commun.

[44] Chen C, Zhang C, Cheng L, Reilly JL, Bishop JR, Sweeney JA, Chen HY, Gershon ES, Liu C (2014). Correlation between DNA methylation and gene expression in the brains of patients with bipolar disorder and schizophrenia. Bipolar Disord.

[45] Lin Q, Weidner CI, Costa IG, Marioni RE, Ferreira MR, Deary IJ, Wagner W (2016). DNA methylation levels at individual age-associated CpG sites can be indicative for life expectancy. Aging (Albany NY).

[46] Horvath S (2013). DNA methylation age of human tissues and cell types. Genome Biol.

