# Supplementary figures and images for: Global DNA methylation changes spanning puberty are near predicted estrogen-responsive genes and enriched for genes involved in endocrine and immune processes
Source: Clin Epigenetics. 2018 May 9;10:62. doi: 10.1186/s13148-018-0491-2 (PMC5941468; doi:10.1186/s13148-018-0491-2)

Beta values using European American samples only (N=50 pairs)

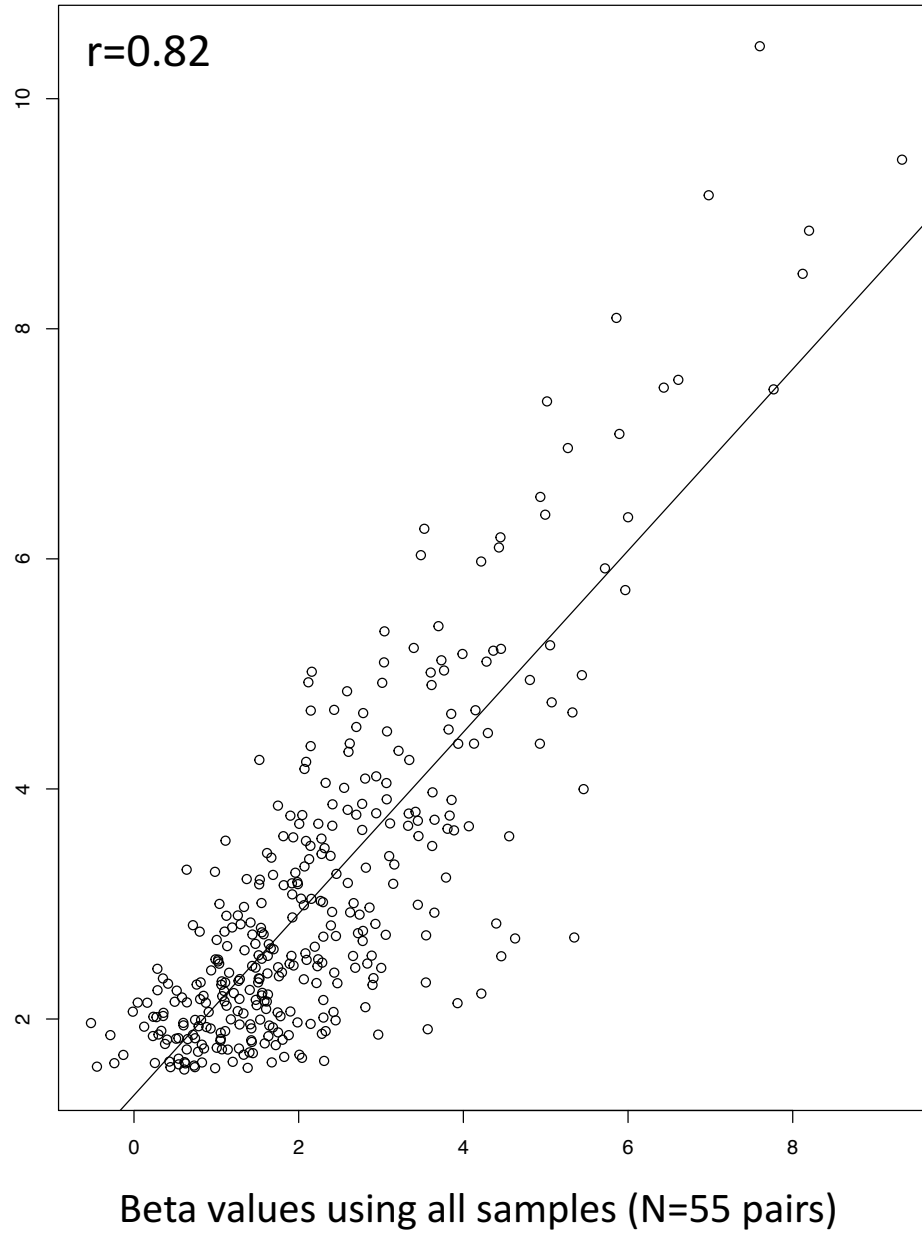

Supplement: Supplementary file 1 — Scatterplot illustrating correlation between methylation beta values in all samples (N = 55 pairs) and beta values following analysis in subjects of European ancestry only. Beta values of 347 DMPs in females from the full sample are plotted on the x axis and beta values from the same DMPs in an analysis using only samples of European ancestry are plotted on the y axis. (PDF 41 kb) [file 13148_2018_491_MOESM1_ESM.pdf]
