# Supplementary material for: Global DNA methylation changes spanning puberty are near predicted estrogen-responsive genes and enriched for genes involved in endocrine and immune processes
Source: Clin Epigenetics. 2018 May 9;10:62. doi: 10.1186/s13148-018-0491-2 (PMC5941468; doi:10.1186/s13148-018-0491-2)
Supplement: Supplementary file 2 — Genes in modules 1 and 2 (as identified by WGCNA) are enriched for predicted estrogen-responsive genes. WGCNA = Weighted gene correlation network analysis. P values reflect results of permutation testing as described in Methods. (DOCX 67 kb) [file 13148_2018_491_MOESM2_ESM.docx]

Additional file 2. Genes in modules 1 and 2 (as identified by WGCNA) are enriched for predicted estrogen-responsive genes (*P*<2.0x10^-6^ by permutation for both tables).

|  | No. of Genes with High Affinity Estrogen Responsive Elements (%) | No. of All Other Genes (%) | Total |
| --- | --- | --- | --- |
| Module 1 | 53^a^ (26.8) | 145 (73.2) | 198 (100) |
| Not in Module 1 | 3,444 (14.7) | 19,987 (85.3) | 23,431 (100) |
| Total | 3,497 (14.8) | 20,132 (85.2) | 23,629 (100) |

| ^a^ Genes in Module 1 that also have high affinity estrogen response elements include:  HOOK2  LAG3  PTPRE  ZNF319  S100A6  MTF1  MAP6  USP15  PPFIBP2  DLX4  ANKRD9  PSMD12  NKD1  SCARB1  SAP30  LCK  GRN  PGLS  LRP5  CYFIP2  COL5A1  LGALS1  TRIM26  DPM2  FKBP14  TCF7  PREP  GAS7  STAT3  MMP23B  UTRN  TPI1  PART1  FXYD6  AXIN2  COMT  CTSD  PACS1  ADAM19  ID3  GLS  SYTL3  CD81  PTPRA  FYN  CYB561  SPRY1  TRPM1  FSD1  TM7SF2  GPC2  IRS2  RNASEH2A   \|  \| No. of Genes with High Affinity Estrogen Responsive Elements (%) \| No. of All Other Genes (%) \| Total \| \| --- \| --- \| --- \| --- \| \| Module 2 \| 20^a^ (23.3) \| 66 (76.7) \| 86 (100) \| \| Not in Module 2 \| 3,477 (14.8) \| 20,066 (85.2) \| 23,543 (100) \| \| Total \| 3,497 (14.8) \| 20,132 (85.2) \| 23,629 (100) \| |
| --- | --- | --- | --- | --- | --- | --- | --- | --- | --- | --- | --- | --- | --- | --- | --- | --- |
| ^a^ Genes in Module 2 that also have high affinity estrogen response elements include:  GRM2  LRAT  RUNX2  ADORA3  LMNA  CHST3  LASP1  WNT6  PECR  FHL2  SMARCB1  LTBP3  SNAPC2  AGXT  STK11  HOXC11  HEYL  CHAF1B  SEC23B  NGB |

|  |
| --- |
|  |
|  |
|  |
|  |
|  |
|  |
|  |
|  |
|  |
|  |
|  |
|  |
|  |
|  |
|  |
|  |
|  |
|  |
|  |
|  |
|  |
|  |
|  |
|  |
|  |
|  |
|  |
|  |
|  |
|  |
|  |
|  |
|  |
|  |
|  |
|  |
|  |
|  |
|  |
|  |
|  |
|  |
|  |
|  |
|  |
|  |
|  |
|  |
|  |
|  |
|  |
|  |
