# Supplementary material for: Global DNA methylation changes spanning puberty are near predicted estrogen-responsive genes and enriched for genes involved in endocrine and immune processes
Source: Clin Epigenetics. 2018 May 9;10:62. doi: 10.1186/s13148-018-0491-2 (PMC5941468; doi:10.1186/s13148-018-0491-2)

Network 1

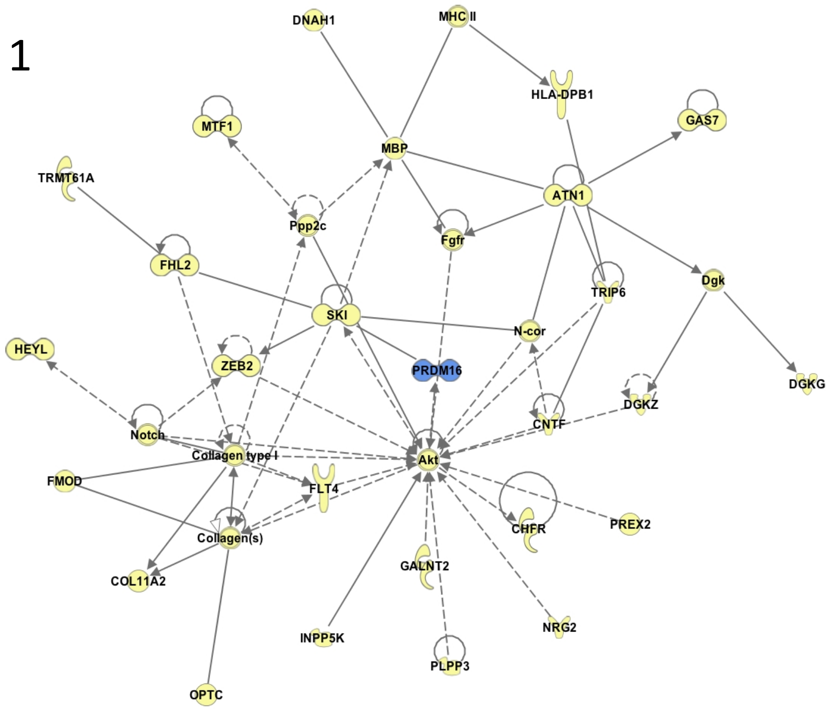

Network 2

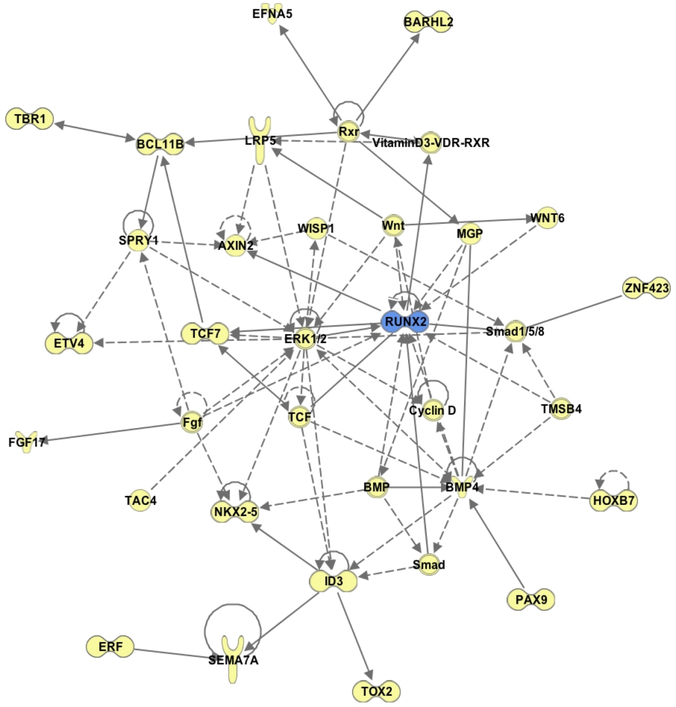

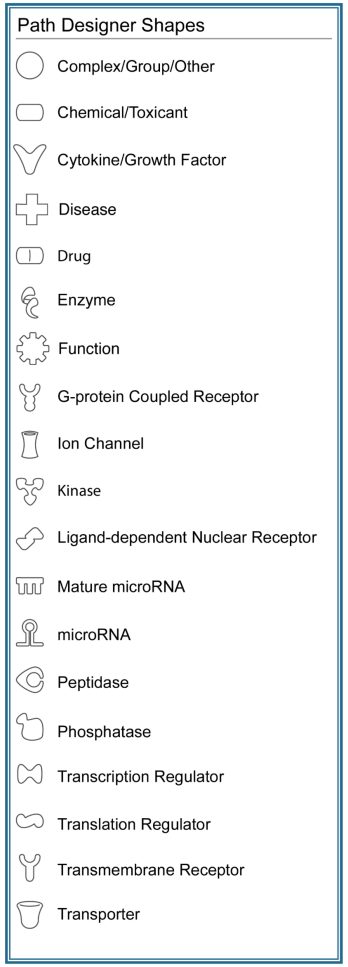

Network 3

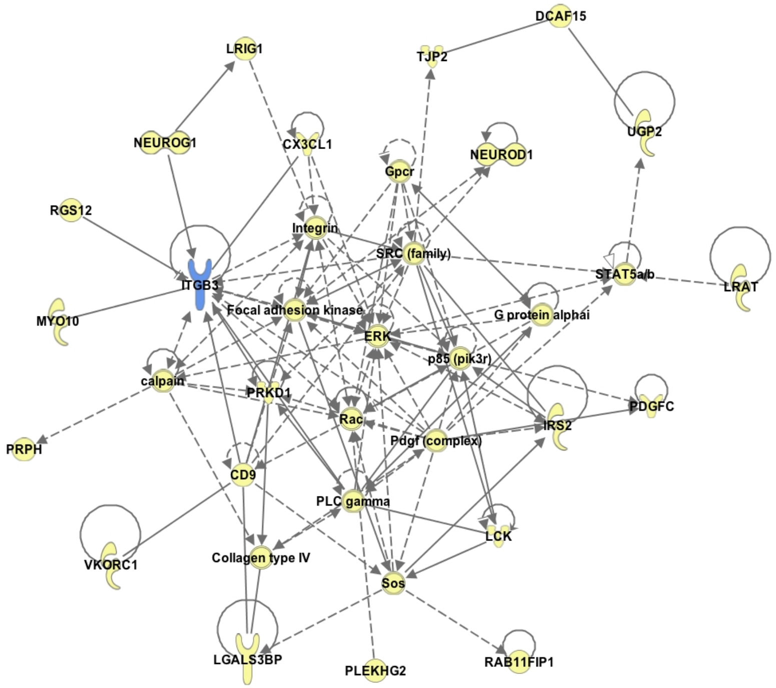

Network 4

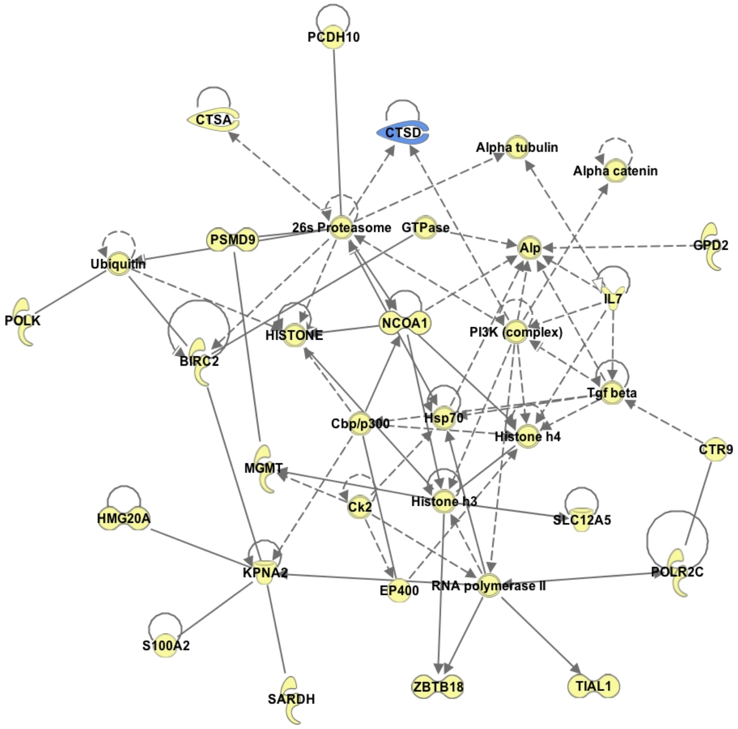

Supplement: Supplementary file 7 — Significant networks of genes associated with female puberty-associated DMCs (from Ingenuity Pathway Analysis). Network 1 score = 47; network 2 score = 41; network 3 score = 33; network 4 score = 32. Genes in blue are shown in more detail in Fig. 2. Shapes shaded in yellow represent genes present in the input list. Solid lines indicate direct interactions between molecules and dotted lines indicate indirect. Arrows indicate the activation of one molecule by another. Legend from https://www.qiagenbioinformatics.com/products/ingenuity-pathway-analysis/. (PDF 3162 kb) [file 13148_2018_491_MOESM7_ESM.pdf]
